# Response to: Potential impact of NK-cell education on interferon efficacy in the ENDURE trial

**DOI:** 10.1038/s41375-026-02985-4

**Published:** 2026-05-26

**Authors:** Andreas Burchert, Christian Michel

**Affiliations:** Department of Hematology, Oncology and Immunology, Carreras Leukemia Center Marburg, University Hospital Giessen and Marburg, Philipps University Marburg, Marburg, Germany

**Keywords:** Medical research, Diseases

## To the Editor

We thank Fujita and colleagues for their thoughtful correspondence [1] regarding the randomized phase 3 ENDURE trial [2] evaluating ropeginterferon alfa-2b as maintenance following tyrosine kinase inhibitor (TKI) discontinuation in chronic myeloid leukemia (CML). We appreciate their recognition of the trial’s prospective design and agree that the overall neutral result is informative for the field, particularly as multiple strategies are being explored to improve the durability of treatment-free remission (TFR) in the contemporary TKI era.

Fujita et al. raise an important biological consideration: interferon’s anti-leukemic activity is at least partly mediated through immune effector mechanisms, including natural killer (NK) cell activation [3], and NK-cell responsiveness is shaped by immunogenetic “education,” notably via the KIR3DL1–HLA-Bw4 axis [1]. We acknowledge that such host factors could, in principle, influence the likelihood of benefiting from interferon-based approaches and may help to explain heterogeneous responses across individuals and populations. Their prior work linking KIR/HLA profiles to depth of molecular response and TFR is compelling and provides a clear rationale for incorporating immunogenetic stratification into future translational efforts [4–6].

At the same time, the key conclusion of ENDURE remains that, in an unselected population meeting the trial’s eligibility criteria, ropeginterferon maintenance did not improve the primary endpoint compared with standard follow-up after TKI discontinuation [2]. As the authors correctly note, ENDURE did not mandate high-resolution KIR and HLA ligand typing and was not designed or powered to test treatment–immunogenetic interactions. Post hoc subgroup exploration—while potentially hypothesis-generating—must be approached cautiously due to multiplicity and the risk of spurious findings, particularly when the biologically relevant subgroup may represent only a minority of patients.

We nevertheless agree with the central message of the correspondence: the negative overall outcome should not be interpreted as definitively excluding a role for interferon in sustaining TFR for all patients. Rather, it reinforces the need for more precise, mechanism-informed approaches. Prospective studies that predefine immunogenetic strata (including HLA-Bw4 status and subtype, and KIR3DL1 allotypes) and pair these with functional immune readouts at the time of discontinuation (e.g., NK-cell phenotype and cytokine responsiveness) would be well positioned to determine whether a clinically meaningful benefit exists in a biologically defined subset. Such designs would also allow appropriate statistical testing for interaction effects, which is essential before translating subgroup signals into practice.

We thank Fujita and colleagues for advancing this discussion and for highlighting a promising direction toward precision immunomodulation in CML. We hope their comments stimulate further collaborative, translationally integrated studies to refine patient selection and optimize strategies aimed at achieving durable TFR.
